# Cervical Spinal Cord Ischemic Reperfusion Injury: A Comprehensive Narrative Review of the Literature and Case Presentation

**DOI:** 10.7759/cureus.28715

**Published:** 2022-09-03

**Authors:** Abdulhadi Y Algahtani, Mouaz Bamsallm, Khalid T Alghamdi, Moajeb Alzahrani, Jehad Ahmed

**Affiliations:** 1 King Abdullah International Medical Research Centre, King Saud Bin Abdulaziz University for Health Sciences College of Medicine, Jeddah, SAU; 2 King Abdulaziz Medical City, National Guard Health Affairs, Jeddah, SAU; 3 Neurosurgery, King Abdulaziz Medical City, National Guard Health Affairs, Jeddah, SAU; 4 Neurosurgery Department, Aseer Central Hospital, Ministry of Health, Abha, SAU

**Keywords:** cervical spinal cord injury, cervical spine transient deficits, white cord syndrome, cervical spine reperfusion injury, cervical cord ischemic injury

## Abstract

Cervical spinal ischemic reperfusion injury (CSIRI) refers to a state of sudden neurological deterioration after surgical spinal decompression. The CSCIRI refers to a state of sudden neurological deterioration after surgical spinal decompression. The pathophysiology is hypothesized to be due to instant relief of a chronically compressed spinal cord, leading to an inflammatory cascade named ischemic reperfusion injury. Deterioration of neurological function after cervical spine decompression surgery often occurs secondary to direct cord injury, compressing hematoma, or hardware failure. Complete loss of neurological function with no organic explanation is an extremely rare complication, with only a few cases reported in the literature. We are reporting a 67-year-old male patient diagnosed with severe cervical spinal canal stenosis at level C5/6 who underwent anterior cervical discectomy and fusion (ACDF). The patient developed complete transient loss of neurological functions after the surgery and was labeled as a case of CSCIRI after excluding compressing pathology. A literature review of the CSCIRI was carried out, and ten articles were included. Due to the rarity of these cases, there is no class 1 or 2 evidence to establish management protocol nor identifiable risk factors to predict their occurrence. However, we recommend using an intra-operative neurophysiology monitor in cases with long-standing severe cervical canal stenosis with myelomalacia and managing these cases according to the acute spinal cord injury management protocol after excluding compressing pathologies.

## Introduction

Cervical spondylosis is one of the most common pathologies of the cervical spine, particularly in geriatric patients. Symptoms may range from paresthesia and lack of dexterity to limb weakness. Cervical spine decompression surgery is one of the most common surgical spine procedures. This type of intervention is considered safe, has a high success rate, and improves functional status in most patients (>80%) [1]; however, it is not without risk. These risks are, to some extent, directly proportional to the magnitude of cervical canal stenosis and the chronicity of the symptoms. Inadequate decompression, epidural hematoma, or direct injury to the spinal cord during surgery are the most common causes of immediate postoperative neurological dysfunction. Acute neurological paralysis after cervical spine decompression without compression factors is extremely rare, with only a few cases reported in the literature. Cervical spinal cord ischemic reperfusion injury (CSCIRI) refers to a sudden deterioration in neurological functions with the appearance of high signal intensity on a T2 magnetic resonance imaging (MRI) sequence. It is a diagnosis of exclusion; Chin et al. described the first case and labelled it as white cord syndrome (WCS) [2]. Neither factors that would increase the rate of its occurrence nor appropriate intervention have been determined. This study is a comprehensive narrative review of the literature with an additional CSCIRI case.

## Case presentation

**Figure 1:**
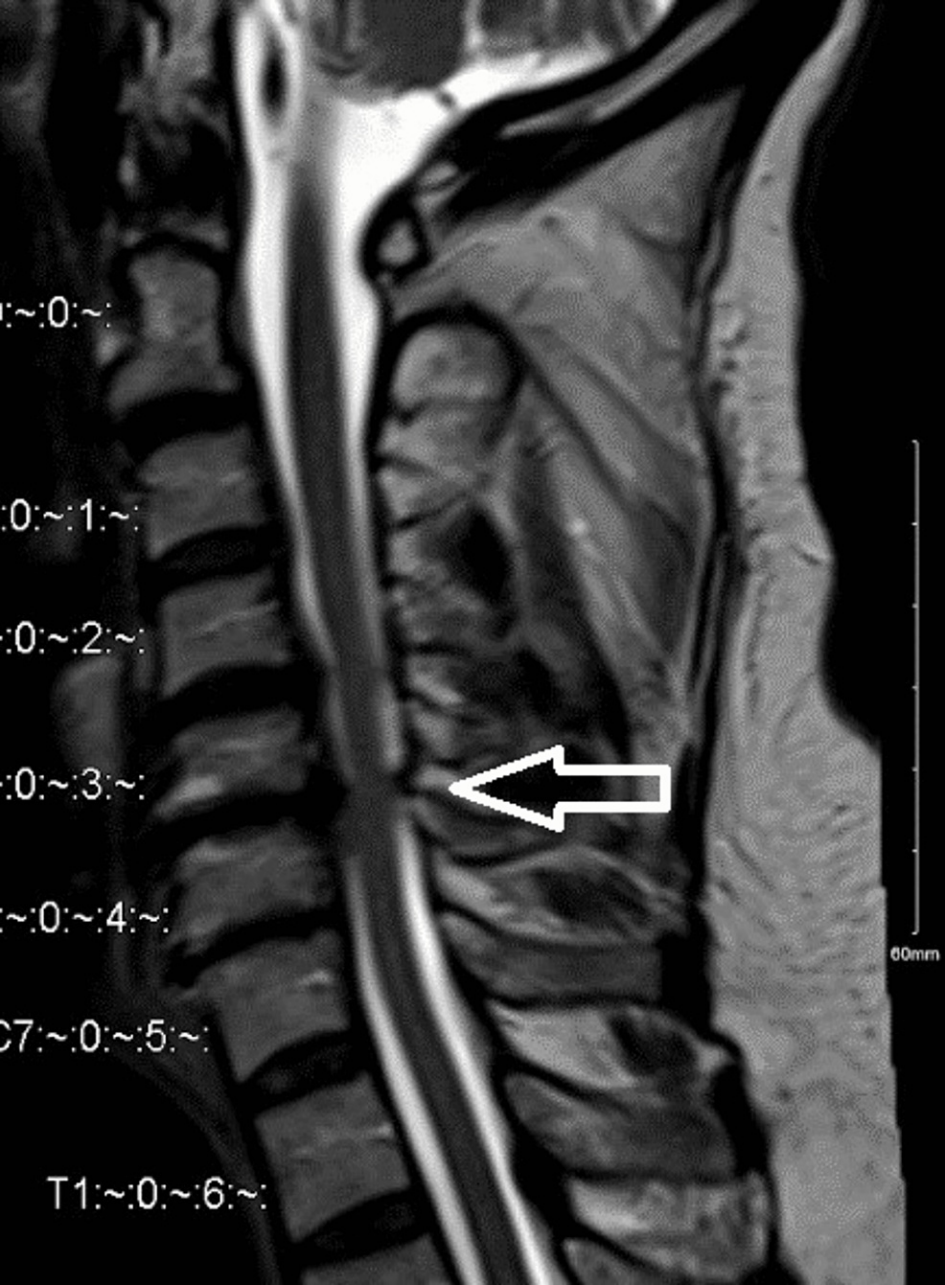
Magnetic resonance imaging scan sagittal view. Revealed multiple level of cervical spine degenerative changes more at C5/6 causing sever canal stenosis and myelomalacia.

A 66-year-old man with severe neck pain of four months' duration radiating to both upper limbs, associated with upper limb numbness, gait disturbance, and urinary urgency. His past medical history included hypertension and ischemic heart disease. A neurological examination revealed normal bulk and tone in all muscle groups. On the modified Medical Research Council scale, motor power in the upper limb was as follows: right shoulder abduction, ⅗; elbow flexion, ⅘; extension, ⅘; wrist and hand grip, ⅘. Left shoulder abduction, ⅗; elbow flexion, ⅘; elbow extension, ⅘; wrist flexion, extension, and hand grip, ⅘. Motor power was 5/5 in all muscle groups of the lower limbs. He had intact reflexes, upper limb paresthesia, hypesthesia, and a positive Hoffman’s sign bilaterally. Pre-operative MRI (Figure 1) demonstrated a large bulge osteophyte complex at the C5/6 level with superimposed central disk extrusion migrating inferiorly.

**Figure 2:**
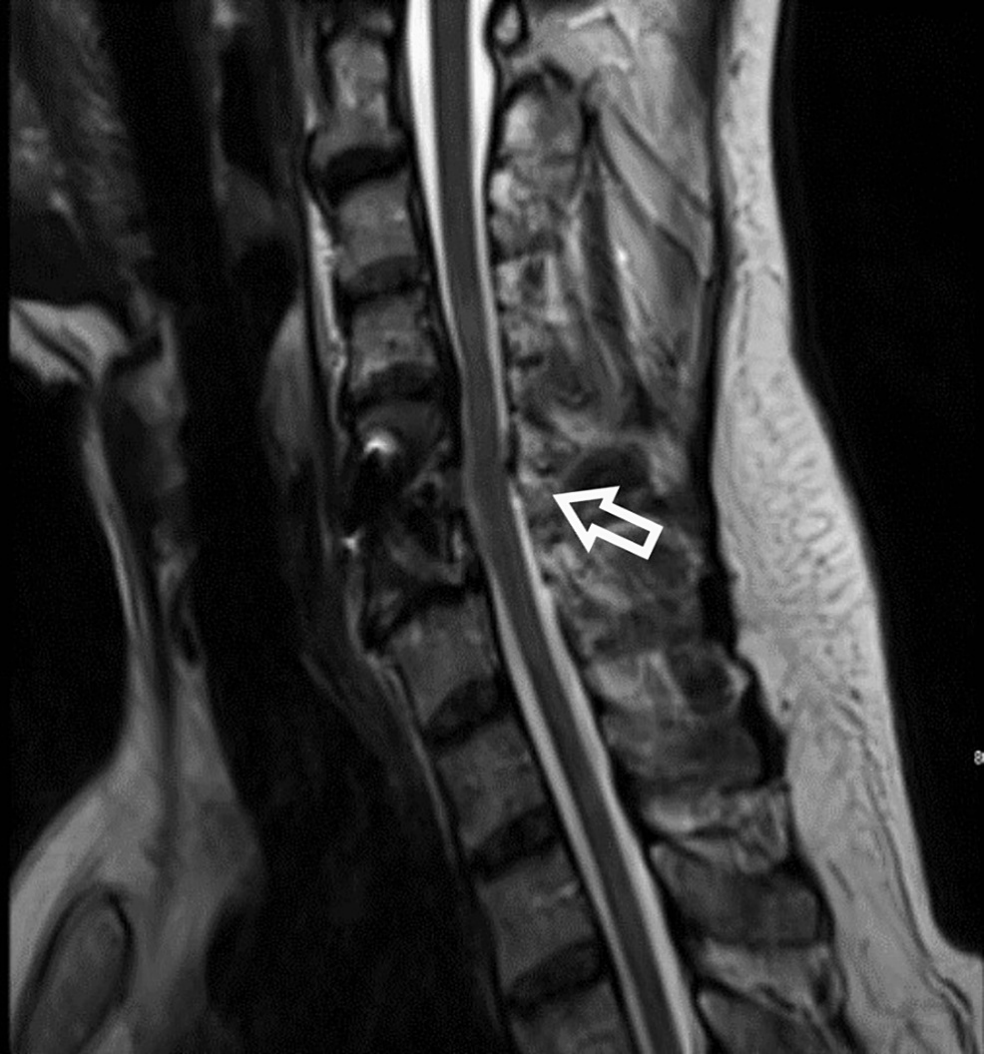
Magnetic resonance imaging sagittal view post anterior cervical discectomy and fusion at level C5/6. Immediate post-op magnetic resonance imaging scan showed no compressing pathology and good spinal decompression however there is evidence of spinal cord expansion and swelling.

The patient underwent anterior cervical discectomy and fusion (ACDF) at C5/6, and no intra-operative complications were observed. The postoperative examination revealed no obvious signs of neurological weakness, and the patient could move all his limbs easily. One hour after the surgery, the patient developed rapidly progressive weakness in all extremities until he became completely tetraplegic. An urgent computed tomography (CT) revealed good cage positioning, no hematoma, and no pathology that would explain the clinical picture. The patient was administered a dose of dexamethasone (10 mg) intravenously and was sent for an MRI scan. Postoperative MRI (Figure 2) showed a slight spinal cord expansion and swelling at the level of C5/6 and no hematoma or collection with interval improvement of the canal stenosis.

**Figure 3:**
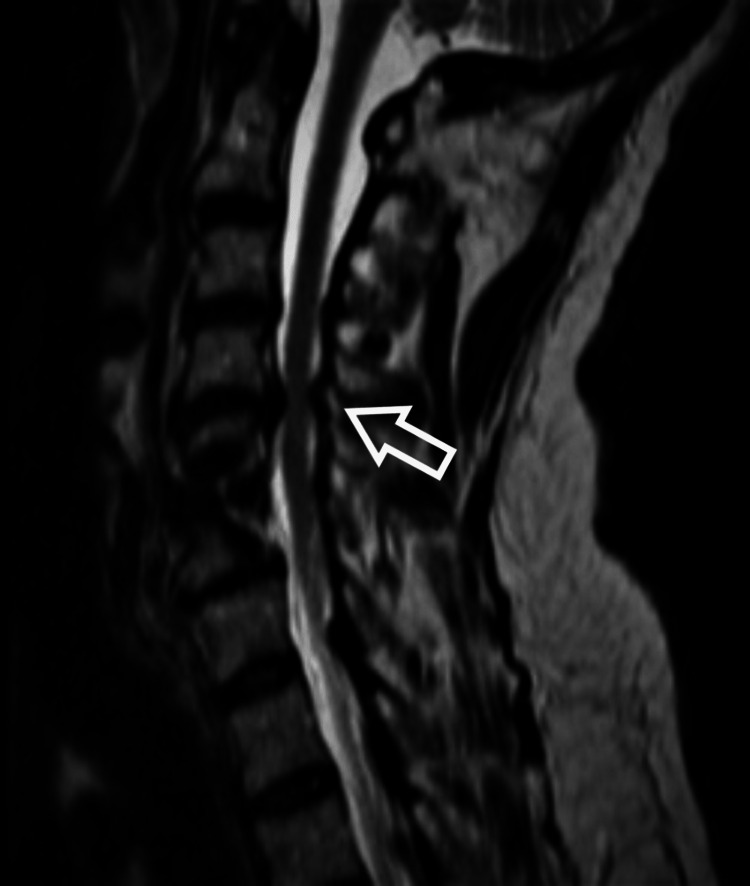
Magnetic resonance imaging scan sagittal view showed stenosis at C4/5. Magnetic resonance imaging scan after six moths showed worsening of cervical stenosis at level above proximal junction level C4/5.

After two hours after the incident, he rapidly regained his motor function. The motor examination showed a power of 5/5 in all the muscle groups. The patient was labeled as a case of cervical spinal cord ischemic reperfusion injury (CSCIRI) after excluding all compressing pathologies. He was kept on dexamethasone 4 mg intravenous every six hours with a mean arterial pressure (MAP) target of 90-100 mmHg. He was discharged home after three days without any motor weakness or deterioration in his neurological function compared to pre-operation. At the first postoperative clinic visit, the patient continued to show full power strength in all muscle groups, with no improvement in sensory function. After six months, he again complained of neck pain, numbness, and urinary incontinence with no motor weakness. Magnetic resonance imaging showed a new severe spinal canal stenosis at level C4/5 (Figure 3).

**Figure 4:**
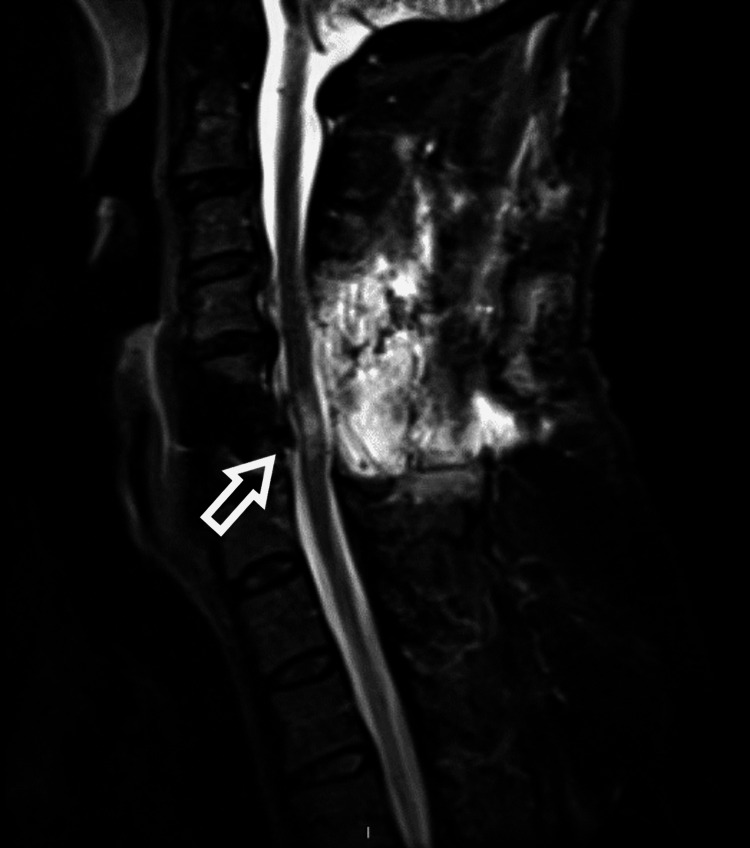
Magnetic resonance imaging scan sagittal view, post posterior laminectomy showed signal changes at C5/6. Magnetic resonance imaging scan revealed the high signal changes at the level C5/6 (at level of cord swelling) after the second surgery. The posterior spinal decompression provides the spinal cord enough space to demonstrate signal changes from previous surgery.

The patient underwent posterior cervical decompressive laminectomy at C3-6. Postoperatively, the patient had normal motor function but there was no significant improvement in sensation. Magnetic resonance imaging (Figure 4) after the second surgery clearly demonstrated a high signal change in the spinal cord at the C5/6 level.

## Discussion

CSCIRI is a recently described neurological condition that may affect patients who undergo cervical spine surgery. Chin et al. reported it for the first time when a 59-year-old man was offered ACDF at C4-6. On an MRI scan, the postoperative patient developed incomplete tetraplegia with an enlarged T2 hyperintense signal. The Chin’s case was then followed up by multiple reported cases worldwide; all of them had the white cord sign appearing or enlarged on MRI postoperatively [2]. The term "white cord syndrome" appeared after excluding all causes that could result in such an event, including incomplete decompression of neuronal elements, instrumental failure, or compressing hematoma [3].

Literature review

**Figure 5:**
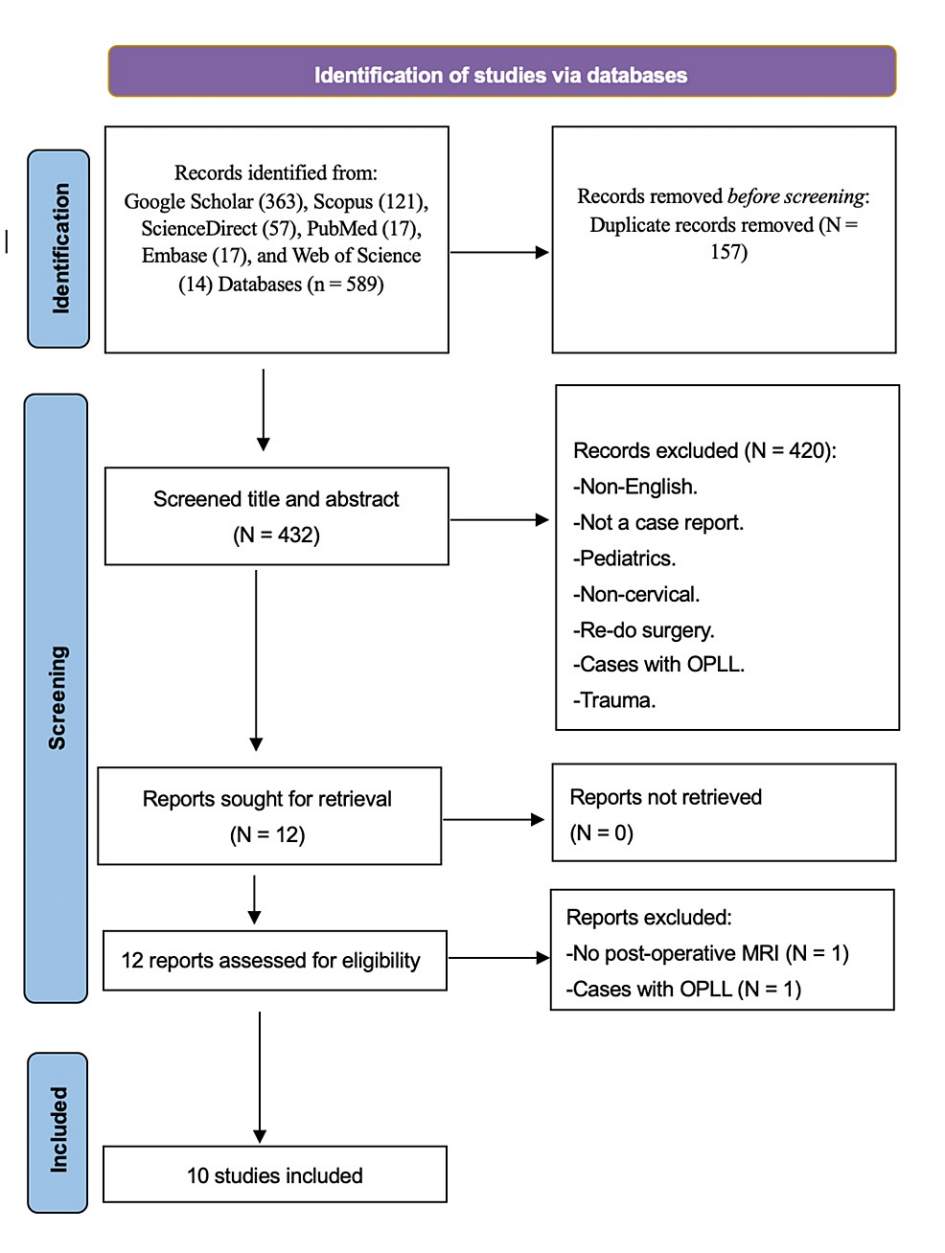
PRISMA search flow diagram. The literature review done according to PRISMA guidelines. Ten articles were included based on inclusion and exclusion criteria. OPLL: ossification of posterior longitudinal ligament, N: number.

A systematic review of the literature in accordance with Preferred Reporting Items for Systematic Reviews and Meta-Analysis (PRISMA) guidelines was conducted by two independent investigators, MB and KA, and any disagreement was resolved by AA [4]. A thorough search of PubMed, Google Scholar, ScienceDirect, Embase, Scopus, Web of Science, and Cochrane databases was performed in January 2022 using ("White cord syndrome" OR "reperfusion injury") AND ("Cervical decompression" OR "anterior discectomy" OR "posterior discectomy"). The English papers were selected and filtered based on the inclusion and exclusion criteria. Adults who underwent cervical operations and developed WCS were included in the study. Pediatric, trauma, non-cervical, repeat surgeries, cancer, and cases with non-degenerative conditions were excluded (Figure 5).

Ten studies were included in the final analysis, and 17 patients participated in the study, of whom 14 were males and 3 were females (Table 1). The average age of the patients was 61 years, with 79 and 36 years being the oldest and youngest ages, respectively. Five patients did not mention associated clinical comorbidities; however, hypertension was observed in 10/12 patients, and one patient had achondroplasia. Only one patient was reported to have no comorbidity. With regard to the MRI changes, 15 of the patients had MRI high signal changes pre-operatively, while two did not have them. However, new signal changes or enlargement of the previous signal have appeared in all cases postoperatively. The addressed surgical approaches were posterior in 12 patients and anterior in five patients. Intraoperative neurophysiology monitoring was used in five cases, and all of them described the loss of either somatosensory evoked potentials (SSEPs) or motor evoked potentials (MEPs). With regard to the timing of neurological deterioration, 14 patients had immediate postoperative neurological deficits, while three developed delayed neurological deterioration after a normal neurological assessment. All reported cases received steroids as a management plan; however, only one patient had steroids before the surgery, and 14 had a rehabilitation program as part of the treatment plan. The mean follow-up duration was three months. With regards to the Nurick classification, pre-operative measurements are as follows: Nurick 1 (23.5%), Nurick 2 (23.5%), Nurick 3 (41.2%), Nurick 4 (5.9%), and Nurick 5 (5.9%). Last post-operative assessment: Nurick 1 (23.5%), Nurick 2 (0%), Nurick 3 (11.8%), Nurick 4 (52.9%), Nurick 5 (5.9%), and mortality in one patient (5.9%).

**Table 1:** Literature review of cervical spine ischemic reperfusion injury. M: male, F: female, ACDF: anterior cervical discectomy and fusion, PD: posterior decompression, PDF: posterior decompression and fusion, MAP: mean arterial pressure, NM: not monition, NC: no comorbidity.

Author	Year	Age	Gender	Exam	Risk factors	Procedure	Intervention	Timing of deficits	Follow-up
Chin et al. [2]	2013	59	M	Nurick 3	NM	ACDF	Steroid + surgery	Immediate	Nurick 4
Giammalva et al. [5]	2017	64	M	Nurick 3	NM	ACDF	Steroid	Immediate	Nurick 4
Khan et al. [6]	2017	36	M	Nurick 5	NC	ACDF	Steroid	Delayed	Nurick 4
Antwi et al. [7]	2018	68	M	Nurick 1	NM	PDF	Steroid+ high MAP	Immediate	Nurick 4
Wiginton IV et al. [8]	2019	41	M	Nurick 1	NM	PD	Steroid+ high MAP	Immediate	Nurick 1
Mathkour et al. [9]	2020	79	M	Nurick 4	HTN	PDF	Steroid + high MAP	Immediate	Nurick 1
Jun et al. [10]	2020	49	F	Nurick 1	HTN	ACDF	Steroid + surgery	Immediate	Nurick 1
Fathalla et al. [11]	2020	62	M	Nurick 2	HTN, DM	PDF	Steroid	Immediate	Nurick 4
		65	M	Nurick 3	HTN	PDF	Steroid	Immediate	Nurick 4
		70	M	Nurick 3	HTN, DM	PD	Steroid	Immediate	Nurick 4
		61	M	Nurick 1	HTN, DM	PDF	Steroid	Immediate	Nurick 3
		63	M	Nurick 3	HTN	PDF	Steroid	Immediate	Nurick 4
		69	F	Nurick 2	HTN, DM	PD	Steroid	Immediate	Nurick 4
		65	F	Nurick 2	HTN	PD	Steroid	Immediate	Nurick 3
Malinovic et al. [12]	2021	46	M	Nurick 3	Scoliosis, Achondroplasia	PD	Steroid	Immediate	Nurick 5
Muke and John [13]	2021	67	M	Nurick 3	NM	PD	Steroid	Delayed	Died
Our case	2022	66	M	Nurick 2	HTN, IHD	ACDF	Steroid + high MAP	Delayed	Nurick 1

Associated factors

It is believed that the incidence of a new T2 hypersignal change in the spinal cord after surgical intervention is higher than expected. In 2004, a cohort study was conducted on 114 patients who underwent cervical decompressive laminoplasty, and the association between postoperative neurological weakness and changes in cervical MRI was studied. The study showed abnormal expansion of the T2 high-signal intensity in 6.1% of patients, and 25% did not suffer from any symptoms. New signal changes were observed in the most stenotic segment of the cervical column [14]. Factors that would increase the rate of its occurrence have not yet been determined. Our observations support the previous belief that high blood pressure may be a factor that increases the incidence of CSCIRI [7,15]. We also notice that it occurs more in males than in females, and this may shed light on the potential hormonal influence on the reaction of neurons to the inflammatory cascade. Due to the rarity of cases, it is difficult to identify risk factors. However, it is reported more with the posterior approach and myelopathic symptoms. In a mouse model, Vidal et al. demonstrated that delayed decompressive surgery for compressive myelopathy was associated with prolonged elevation of inflammatory cytokines and an exacerbated peripheral monocytic inflammatory response, which led to an increased risk of reperfusion injury. Vidal et al. used six months as a time frame to differentiate between early and late symptoms [1]. A preoperative MRI showed high signal changes in all the patients except for two, which indicated that the spinal cord was severely affected by the stenosis, and myelomalacia may be considered as one of the factors that may increase the probability of reperfusion injury.

Pathophysiology

The mechanism underlying this phenomenon is still not clearly understood; some reports have suggested that spinal shock and reperfusion injury secondary to sudden neuronal decompression are the primary causes of neurological deterioration by initiating inflammatory cascades. Several pathophysiological mechanisms of WCS have been reported in the literature: (i) ischemia-reperfusion injury, which occurs due to direct trauma from blood flow, disturbance in the blood-spinal cord barrier, or the development of free oxygen radicals; (ii) microthrombi, which occlude vascular supply to the watershed regions; and (iii) recoiling shape of the spinal cord due to chronic compression, which leads to changes in perfusion [9,15]. The delayed deterioration of spinal functions after surgical decompression supports the theory of reperfusion ischemic injury, as incomplete spinal decompression or direct trauma to the spinal cord will result in immediate loss of spinal cord functions. In our case, the patient did not show signs of loss of neurological functions until two hours after the operation. The immediate post-op assessment was normal. The same happened in a study by Page et al. [4] and Hussein et al. [11], as the patient developed neurological deficits 72 hours after the surgery. In the study by Epstein [3] and Giuseppe et al. [5], cases developed somatosensory and motor-evoked potential loss during the closure of the fascia.

Interventions and outcome

All patients received high-dose steroid protocols; two underwent additional surgical decompression; and five reported an increase in MAP of >85 mmHg. This effect of steroids on mitigating symptoms may support the claim that the inflammatory cascade is part of the pathophysiology of WCS. Because of the rarity of these cases, there are no evidence-based recommendations to treat them; therefore, it is preferable to use the guidelines for the traumatic spinal cord as in most cases. Managing these cases in an intensive care unit or continuous monitoring unit is advisable to maintain a high MAP and prevent episodes of hypotension or hypoxia. Additionally, imaging, including CT or MRI scans, is recommended to roll out any reversible pathological etiologies [12]. Regarding the outcome, five patients showed remarkable improvement, while 10/17 showed partial improvement. However, the final outcome was worse than the pre-operative status. One patient failed to show any significant improvement, and one died.

Limitations

The study has limitations as all the data were extracted from case series and case reports; therefore, the quality of this study relies on the quality of the included articles. However, considering the extremely rare occurrence of this complication, the article provides the best and most recent evidence of cervical spinal cord reperfusion injury.

## Conclusions

Cervical spine cord ischemic reperfusion injury is a rare, devastating complication that needs to be included as a potential outcome during counseling of patients for surgical decompression in severe cervical spondylosis. Hypertension and pre-operative myelomalacia were the most common associated factors. Therefore, it is recommended to consider using a neurophysiological monitor, especially in cases with long-standing compressive myelopathy, for early detection and establishment of management measures. It is important to call for an in-depth study of the changes that occur in the spinal cord as a result of degenerative spondylosis and for interventions that would mitigate CSCIRI or prevent its occurrence.
